# City-Scale Expansion of Human Thermoregulatory Costs

**DOI:** 10.1371/journal.pone.0076238

**Published:** 2013-10-15

**Authors:** Richard W. Hill, Timothy E. Muhich, Murray M. Humphries

**Affiliations:** 1 Department of Zoology, Michigan State University, East Lansing, Michigan, United States of America; 2 Department of Natural Resource Sciences, McGill University, Ste-Anne-de-Bellevue, Quebec, Canada; University of Regina, Canada

## Abstract

The physiological maintenance of a stable internal temperature by mammals and birds – the phenomenon termed homeothermy – is well known to be energetically expensive. The annual energy requirements of free-living mammals and birds are estimated to be 15–30 times higher than those of similar-size ectothermic vertebrates like lizards. Contemporary humans also use energy to accomplish thermoregulation. They are unique, however, in having shifted thermoregulatory control from the body to the occupied environment, with most people living in cities in dwellings that are temperature-regulated by furnaces and air conditioners powered by exogenous energy sources. The energetic implications of this strategy remain poorly defined. Here we comparatively quantify energy costs in cities, dwellings, and individual human bodies. Thermoregulation persists as a major driver of energy expenditure across these three scales, resulting in energy-versus-ambient-temperature relationships remarkably similar in shape. Incredibly, despite the many and diversified uses of network-delivered energy in modern societies, the energy requirements of six North American cities are as temperature-dependent as the energy requirements of isolated, individual homeotherms. However, the annual per-person energy cost of exogenously powered thermoregulation in cities and dwellings is 9–28 times higher than the cost of endogenous, metabolic thermoregulation of the human body. Shifting the locus of thermoregulatory control from the body to the dwelling achieves climate-independent thermal comfort. However, in an era of amplifying climate change driven by the carbon footprint of humanity, we must acknowledge the energetic extravagance of contemporary, city-scale thermoregulation, which prioritizes heat production over heat conservation.

## Introduction

**Figure 1:**
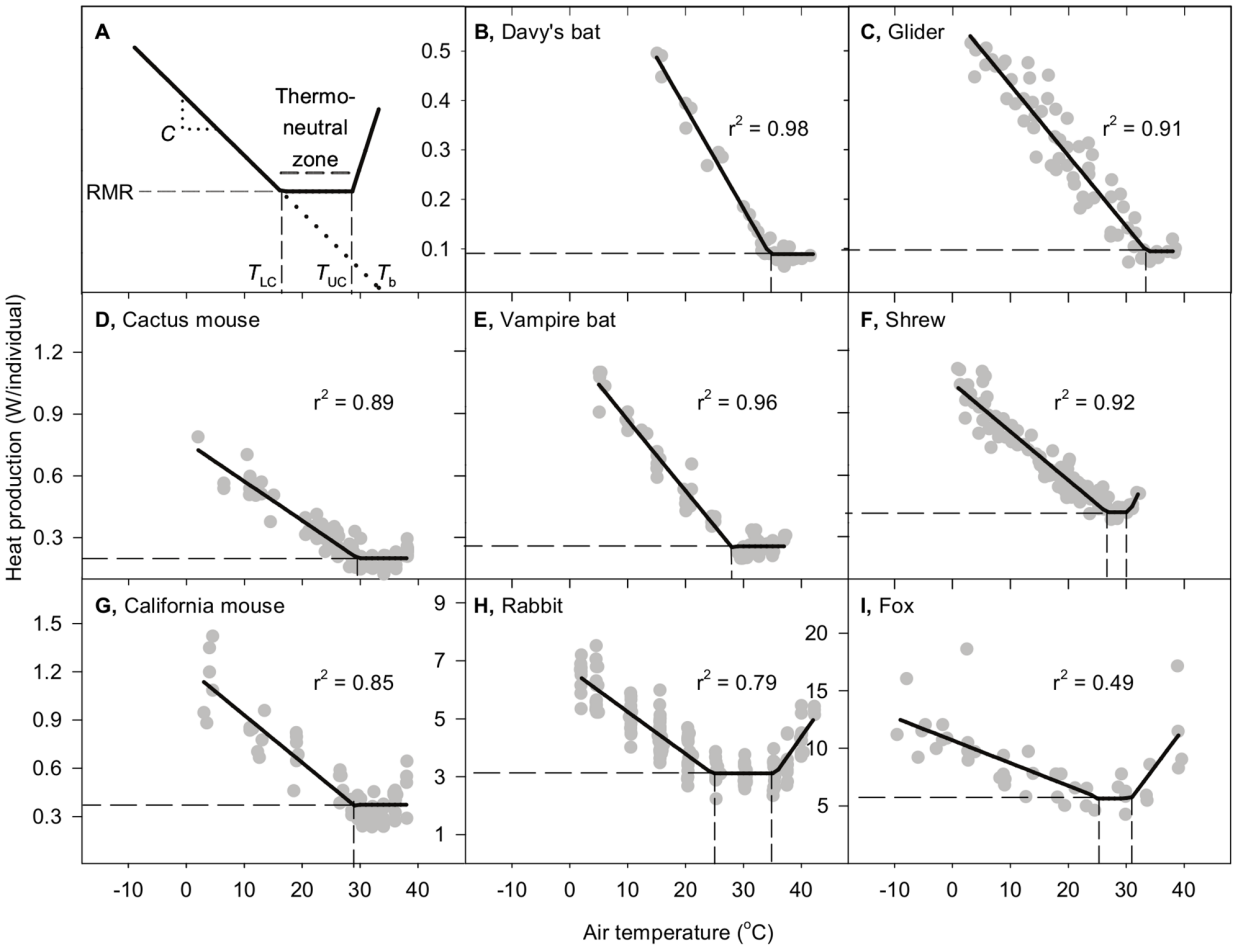
Rate of energy use as a function of ambient temperature (*T*_a_) in mammalian species: interpretive framework and examples. (A) The conventional metabolic rate-*T*_a_ relation in individual homeotherms [1]–[2]. Within the *thermoneutral zone* (*TNZ*), the animal’s metabolic rate, termed its *RMR*, is low and independent of *T*_a_. The lower and upper limits of the TNZ are the *lower* (*T*_LC_) and *upper* (*T*_UC_) *critical temperatures*. Thermoregulation in the TNZ is achieved by autonomic modulation of body insulation: low near *T*_UC_ but high near *T*_LC_. At *T*_a_<*T*_LC_, body insulation is approximately constant, and accordingly the rate of metabolic heat production required for thermoregulation increases as *T*_a_ falls. The absolute value of the slope of this increase is *conductance* (*C*). Based on a first-order model, extrapolation is expected to intersect the abscissa at *T*_a_ equal to body temperature (*T*_b_). (B-I) Metabolic rate-*T*_a_ relations in eight mammal species. Each symbol represents one individual at one *T*_a_. Vertical dashed lines identify *T*_LC_ and *T*_UC_ where statistically defined. Horizontal dashed line identifies resting metabolic rate (RMR). Species and sources are: B, *Pteronotus davyi* [32]; C, *Acrobates pygmaeus* [33]; D, *Peromyscus eremicus* (Nevada) [34]; E, *Desmodus rotundus* [35]; F, *Blarina brevicauda* [36]; G, *Peromyscus californicus* (*parasiticus*) [34]; H, *Sylvilagus audubonii* (winter) [37]; I, *Vulpes macrotis* (winter) [38].

Homeothermic mammals and birds maintain constant body temperatures, independent of air temperature (*T*_a_), by intensifying metabolic heat production as their environment becomes colder and often by using metabolic energy to drive cooling as conditions become hotter [1]–[2]. This results in a characteristic \_/-shaped relationship between rate of energy use and *T*_a_ (Fig. 1A). Explanations for the evolution of homeothermy remain controversial, but often invoke improved performance across a range of climate conditions [3]. Regardless of the benefits of homeothermy, the energy costs of this opulent lifestyle are clear. Based on studies of free-living vertebrates using the doubly-labeled water method, Nagy estimated that the annual energy requirements of free-living homeotherms are 15–30 times higher than those of similar-size ectothermic vertebrates like lizards [4]. As prolific degraders of free energy into heat, homeotherms contribute disproportionately to energy dissipation in natural food webs [5] and greenhouse gas emissions [6].

The implications of human homeothermy in defining the energy demands of contemporary human populations are rarely discussed, possibly because of the indirectness with which modern humans experience thermoregulatory energy costs. Today, most humans living in thermally challenging environments occupy dwellings maintained near thermoneutral temperatures using exogenous energy sources such as natural gas and electricity. With the dwellings often incorporated into urban-industrial systems [7]–[8] dependent on long-distance energy-supply networks, the contemporary human costs of thermoregulation are emergent properties operating at the scale of cities rather than that of individual bodies metabolizing endogenous fuels. Here we analyze daily energy use by cities to determine whether thermoregulation persists as a major driver of energy expenditure in human populations and, if so, its implications for the contemporary energy demands of living.

We hypothesized that cities would have energy-*T*_a_ relationships similar in form to those of individual homeotherms because both are constrained by basic physical principles of heat production and transfer. However, recognizing the confounding effects of non-thermoregulatory energy uses in cities, we expected the strength of the energy-*T*_a_ relationship to be weaker in human cities than individual homeotherms. We also hypothesized that cities would resemble wild homeotherms in showing latitudinal trends toward increased baseline energy expenditure [9]–[10] and improved insulation in colder climates. We tested these hypotheses using data on six mid-size North American cities (population: 25,000–66,000), distributed over a 24°C latitudinal gradient in average temperature, that rely primarily on electricity and natural gas as home energy sources. The six cities used (Ames, IA; Dothan, AL; Flagstaff, AZ; Key West, FL; Kissimmee, FL; and Timmins, Ontario) were those for which we were able to get full data during a survey of 39 cities within the target range of sizes. For each city, we obtained data on daily use of electricity and natural gas for an entire year (either 2009 or 2010, except that for Kississmee the dates were April 1, 2010, to March 31, 2011), permitting the relationship between energy use and *T*_a_ (Table S1) to be examined with daily resolution.

By integrating daily energy use to estimate annual energy use, we have also sought to estimate the cost of what Bateson [11] termed extraregulation. Extending Prosser’s categorization of animal poikilotherms as adjusters and homeotherms as regulators [12], Bateson categorized humans that modify their environment to achieve regulation as extraregulators. Acknowledging that the adjuster-to-regulator transition required a 15-30 fold increase in the annual energy expenditures of vertebrates [4], what is the additional cost of the regulator-to-extraregulator transition? To address this question, we recognize naked individuals, clothed individuals, wood-heated cabins, and cities as logical focal points along a continuum of primordial and derived states in human thermoregulation. Then we synthesize, with our new data on cities, five decades of experiment, observation, and theory to elucidate the similarities and critical distinctions among these grades of human thermoregulatory energetics.

## Results, Discussion, and Conclusions

**Figure 2:**
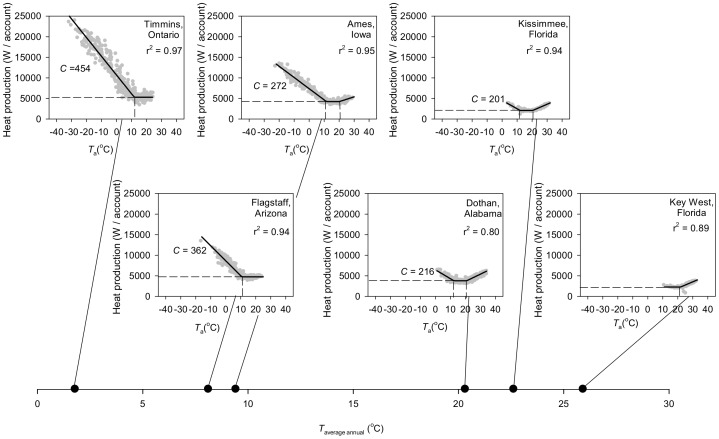
Rate of energy use as a function of ambient temperature (*T*_a_) in six North American cities. Each plot covers 365 days, with each symbol representing one day. The rate of heat production each day is expressed per utility account (i.e., per separately billed house or other living unit), and *T*_a_ is the daily average (Table S1) for the same day. *T*_average annual_ is the average *T*_a_ for all days included in the annual dataset. Lines between individual city *T*_a_ axes and the *T*_average annual_ axis connect equal temperatures. Vertical and horizontal dashed lines are as in Fig. 1A. *C* is the absolute value of the slope at *T*_a_<*T*_LC_ (see Fig. 1A).

Cities (Fig. 2) exhibit energy-*T*_a_ relationships similar in form to those of individual mammals (Fig. 1), a pattern that is also apparent in a plot of aggregated monthly US energy consumption as a function of US nationwide average monthly temperature [13] and that is suggested by the seasonality of natural gas use in the United Kingdom [14]. Of the six cities we have studied, the coldest five show clear lower critical temperatures (*T*_LC_), below which daily energy use increases linearly with declining daily *T*_a_ along a slope defined by conductance *C* (Fig. 1A). The warmest four cities show clear upper critical temperatures (*T*_UC_), above which energy use increases as *T*_a_ rises (expected with air conditioning use).

Contrary to our initial expectation, thermoregulation is as important a driver of energy expenditure at city scale (mean adj-r^2^ for the entire multi-phase regression  =  0.92, range  =  0.80–0.97, *n* = 6 cities) as it is for homeotherms studied alone and inactive in metabolic chambers (Fig. 1B-I; mean adj-r^2^ = 0.85, range  =  0.49–0.98, *n* = 8 species).

Unlike the trend in wild homeotherms, we found no trend for cold-climate cities to have lower *T*_LC_ or lower *C* than warm-climate cities (Fig. 2; Table 1) – pointing to no climatic adaptation in insulation at a city scale in North America. However, city resting metabolic rate, RMR, (Table 1) increases from warmest to coldest. The RMR in the coldest-climate city (5300 W/account) is 2.3-2.5 times that in the two warmest-climate cities (2100–2300 W/account). Thus, whereas wild homeotherms evolutionarily increase both insulation and RMR in response to cold challenge [9]–[10], human cities in North America appear to employ only an energy-demanding RMR response.

**Table 1:** Statistical results for the six cities, listed (left to right) from highest to lowest average annual temperature.

Property	City					
	Key West,Florida	Kissim-mee,Florida	Dothan, Alabama	Ames,Iowa	Flagstaff,Arizona	Timmins,Ontario
*T*_average annual_ (°C)	25.9	22.6	20.3	9.4	8.1	1.8
*T*_LC_ (°C)	—	12.5	12.3	11.5	10.7	11.9
*T*_UC_ (°C)	21.4	21.1	20.8	20.2	—	—
RMR (W account^−1^)	2290	2110	3790	4250	4760	5290
RMR (W person^−1^)	880	810	1460	1630	1830	2030
Slope below *T*_LC_(W°C ^−1^ account^−1^)	—	−201	−216	−272	−362	−454
Slope below *T*_LC_(W°C ^−1^ person^−1^)	—	−77.3	−82.9	−105	−139	−174
Slope above *T*_UC_(W°C^−1^ account^−1^)	153	164	177	126	—	—
Slope above *T*_UC_(W°C ^−1^ person^−1^)	58.8	63.1	68.0	48.5	—	—
Adj-r^2^ (entire multi-phase regression)	0.89	0.94	0.80	0.95	0.94	0.97
Population (persons)	25000	61000	66000	59000	66000	43000
Longitude (°W)	81.8	81.4	85.4	93.6	111.7	81.3
Latitude (°N)	24.6	28.3	31.2	42.0	35.2	48.5
Altitude (m)	6	27	120	280	2100	300

*T*_LC_ and *T*_UC_ are listed only if defined by convergence and significance in multi-phase regression. RMRs and slopes are expressed per utility account (i.e., per separately billed house or other living unit) and per person on the assumption of 2.6 people per account [40].

Direct data on city energy use of a similar sort to ours have not been compiled for other regions of the world. However, a summary of percentages of houses with insulation in European countries suggests that insulation correlates negatively with winter temperature [15]. Traditions of housing construction and economic factors (e.g., cost of heating versus cost of insulating) may differ among world regions. These considerations suggest that an important next step in the study of city-scale thermoregulatory energetics is to do direct energy-use comparisons among world regions.

**Figure 3:**
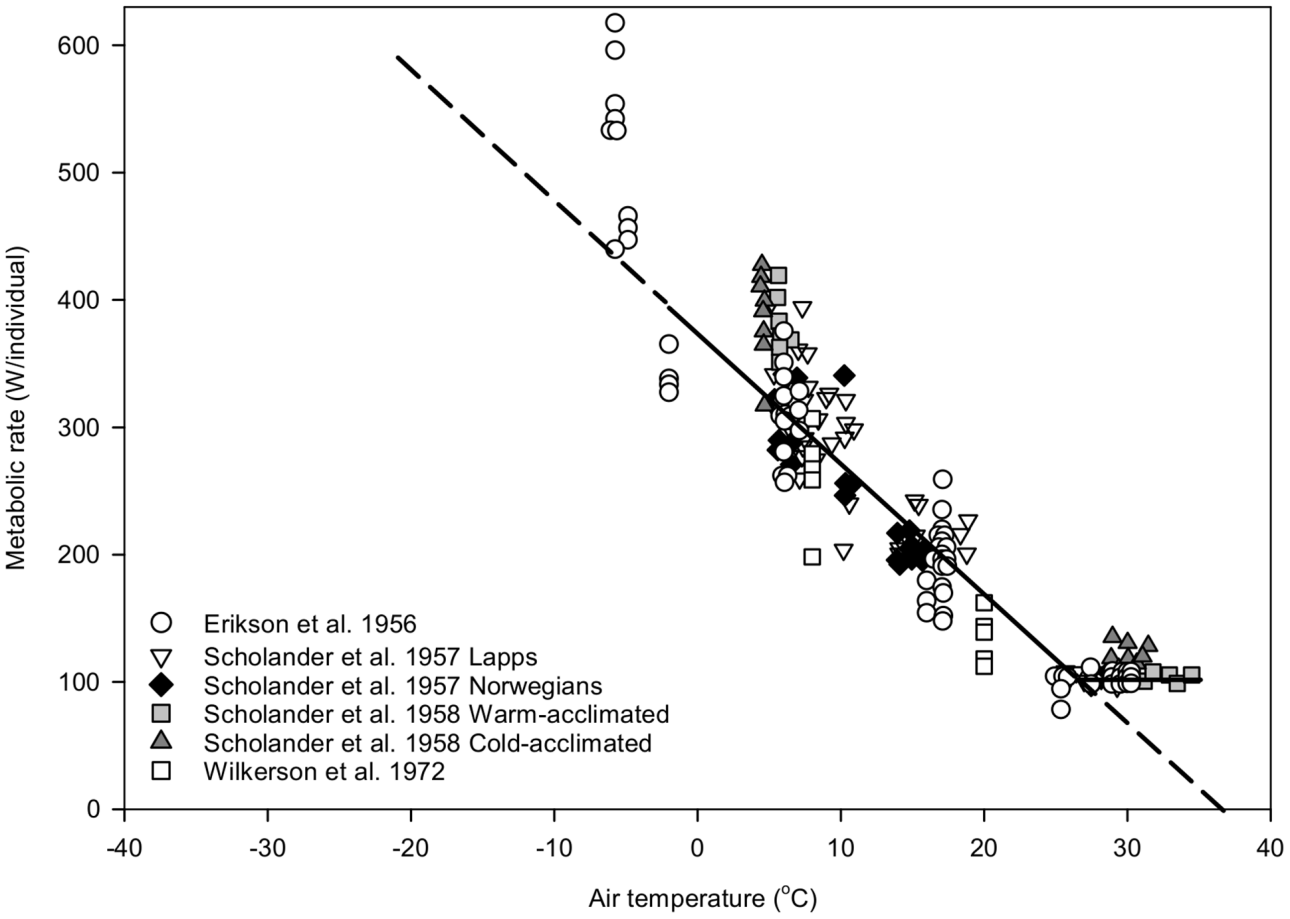
Meta-analysis of metabolic cost of thermoregulation in naked men: metabolic rate per person as a function of ambient temperature (*T*_a_). Men wore skimpy shorts and in some cases shoes (to peddle an ergometer). Values at *T*_a_<−3°C were excluded from the regression analysis because subjects were notably uncomfortable under such cold conditions. Original papers presented metabolic rates as percentages of basal or resting metabolic rates, i.e., 100  =  basal or resting rate, 200  =  twice basal or resting, etc. Linear least-squares regression was carried out in this scaling domain: Metabolic rate as percentage of basal or resting  =  −9.812 (*T*_a_ − 37) (r^2^ = 0.98). For this plot, a 178-cm-tall, 70-kg subject (body surface area  =  1.8 m^2^) [39] with resting metabolic rate  =  1 met (50 kcal m^−2^ h^−1^ = 58 W m^−2^) [20] is assumed. A basal or resting metabolic rate of 104 W is thus assumed: Metabolic rate (W/individual)  =  −10.27 (*T*_a_ − 37). Citations: Erikson et al. 1956 [16]; Scholander et al.1957 [17]; Scholander et al.1958 [18]; Wilkerson et al. 1972 [19].

**Figure 4:**
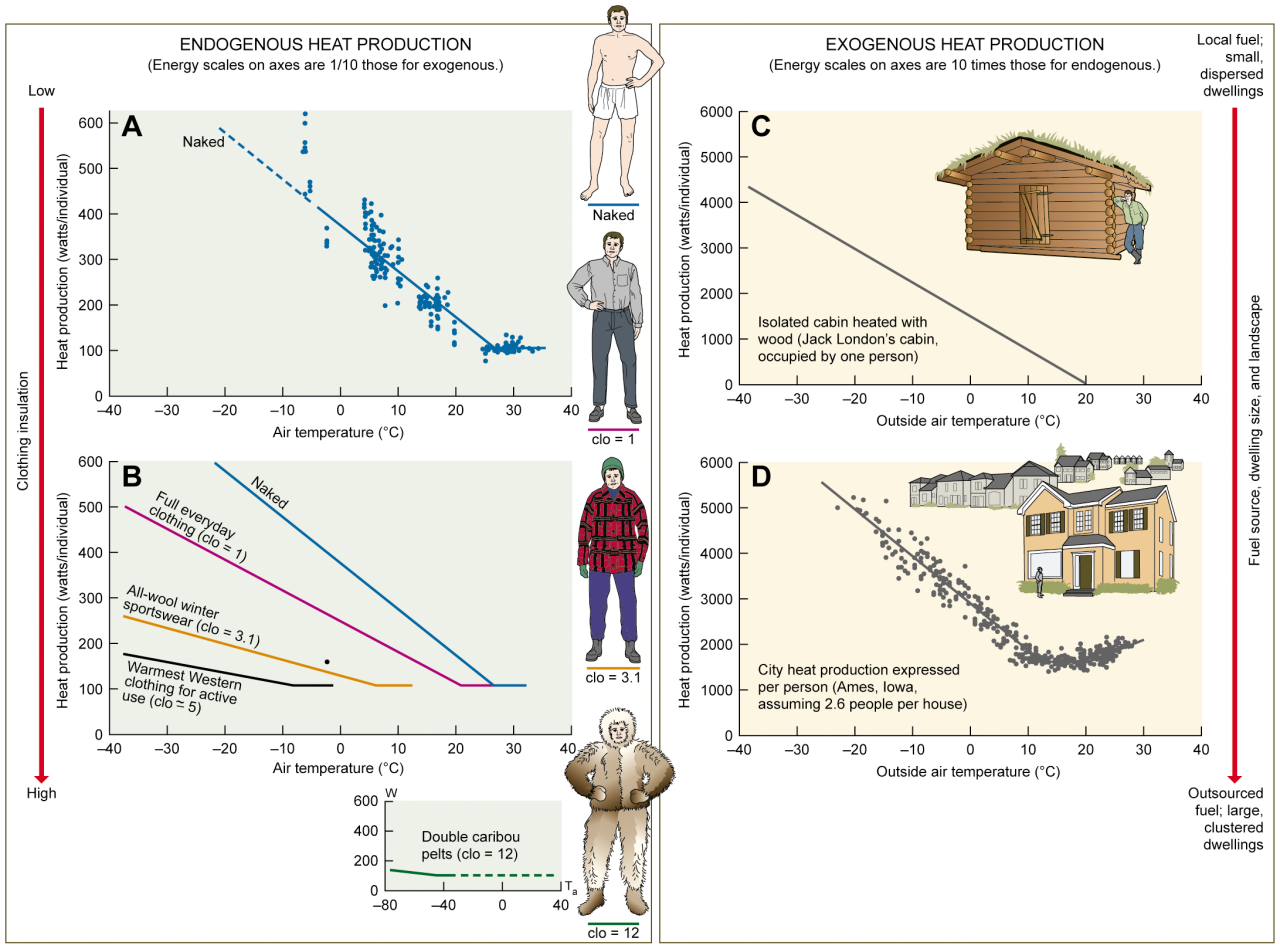
Per capita energy demands of human thermoregulation by endogenous and exogenous heat production. (A) Meta-analysis of all pertinent data available on thermoregulation in naked people from Fig. 3. (B) Metabolism-*T*_a_ curves for clothed people (equations and derivation in Table S2). Only a narrow thermoneutral zone (TNZ) is shown in each plot because a person would not elect to stay in the specified clothing at highly elevated *T*_a_. Dot is a direct measurement on a man in winter sports clothing [16]. (C) The rate of addition of heat to the interior of Jack London’s Yukon cabin required to maintain an interior temperature of 20°C (Table S3): W/individual  =  −74.4 (*T*_a_ – 20°C). A cabin heated with hand-cut wood and devoid of electricity has no TNZ because the fire would be allowed to go out at high outside *T*_a_. (D) Data from Fig. 2 for Ames, IA, a city with median properties, assuming 2.6 people per household [40]. Regression below thermoneutrality: W/individual  =  2863 – 105*T*_a_.

Individual naked humans can thermoregulate at moderate to low *T*_a_ entirely through metabolic heat production [16]–[19]. Our meta-analysis of previously unsynthesized studies (Fig. 3; Fig. 4A) indicates that individual naked humans begin to elevate metabolism above a RMR of about 104 W when *T*_a_ drops below a *T*_LC_ of 26–27°C, along a slope defined by *C* = 10.3 W/°C. By donning clothing of increasing insulative value, people can progressively reduce *T*_LC_ and *C*. Here, by using the fundamental equations for clo calculations [20]–[21], we model the energy-*T*_a_ relationships of clothed humans using clo values [22]–[23] that quantify the insulative values of clothing types (Table S2; Fig. 4B). Typical indoor clothing (clo  = 1) reduces *T*_LC_ to 21°C and *C* to 6.5 W/°C, whereas the warmest Western clothing practical for activity (clo  =  5) extends these to −7°C and 2.4 W/°C. Traditional Inuit of Alaska and Sami of Norway wore the most insulating clothing ever measured [17], [22]; their double caribou pelt garments (clo  =  12) [22] would have reduced *T*_LC_ to about −50°C and *C* to 1 W/°C.

Over broad spans of space and time, the temperature-controlled dwellings occupied by humans have been minimalist structures – such as animal-pelt tents, igloos, and mud-brick houses [24] – providing only a small heated living space per person. Fuel was often locally collected wood. We use the small (3.5×3.5×2.7 m inside), wood-heated log cabin in which Jack London lived alone during the winter of 1897-1898 in the Yukon as a representative minimalist dwelling. Combining historical records of this cabin’s construction [25] with standard insulation analysis for individual dwellings [26]–[28], we estimate (Table S3) that maintaining this cabin at an inside temperature of 20°C would have required a rate of internal heat addition escalating from 0 W at *T*_a_ = 20°C to 1500 W at 0°C and to 5200 W at −50°C, an extreme low temperature that occasionally occurs in the Yukon (Fig. 4C). As is well documented for medieval London, England [29], population growth eventually causes wood use to exceed local forest productivity, necessitating an expanding network of fuel production and distribution. In modern cities in places like North America, dwellings provide a large climate-controlled living space per person and depend on sprawling energy distribution networks. Ames, IA, a median North American city, has per capita heating costs of 1600 W when *T*_a_ = 20°C, increasing to 2900 W at 0°C and 8100 W at −50°C (Fig. 4D).

Overall, the fundamental form of the energy-*T*_a_ relationship persists among naked and clothed humans using endogenous fuels, and among both minimalist dwellings and modern cities using exogenous fuels (Fig. 4). The critical differences are the substantial reduction in *T*_LC_ and *C* offered by insulating clothing (Fig. 4B versus Fig. 4A) and the substantial increase in energy use associated with the transition from endogenous to exogenous fuels (Fig. 4C–D versus Fig. 4A–B).

To estimate the cost of extraregulation [11], we ask: Under long-term, realistic *T*_a_ scenarios, what is the cost of human thermoregulation in exogenously fueled, climate-controlled dwellings relative to the cost of an entirely endogenous, metabolic existence? One approach to answering this question is to focus on locales such as Dothan, Key West, and Kissimmee where a naked, metabolic existence is conceivable. Focusing, therefore, on those three cities, we calculated annual energy costs of people living indoors (Fig. 2) and naked outdoors (Fig. 3; Fig. 4A) using decadal *T*_a_ records for the years 2000–2009. The per capita annual cost of indoor, exogenous thermoregulation (57, 36, 32 GJ in Dothan, Key West, and Kissimmee, respectively) averages 9 times the cost of endogenous thermoregulation (5.7, 4.0, 4.8 GJ). An alternative approach, applicable to locales with cold winters (e.g., Ames, Flagstaff, and Timmins), is to contrast the energy costs of people living indoors (Fig. 2) and clothed outdoors (Fig. 4B). By adjusting clothing to match *T*_a_, a person can maintain a RMR of about 104 W regardless of *T*_a_ (Fig. 4B), corresponding to an annual cost of thermoregulation of 3.3 GJ. In Ames, Flagstaff, and Timmins, the annual cost for a person living alone in a steadily warmed cabin like Jack London’s (Fig. 4C), based on decadal *T*_a_ records, averages 10 times higher (27, 27, 41 GJ in the 3 cities), while for a person in a city house (Fig. 4D) the annual per-person cost averages 28 times higher (72, 78, 122 GJ; ratio calculated before rounding). This stunning 28-fold increment arises from city costs, of course, but also from the impressive energy conservation that an isolated individual can achieve by adjusting clothing insulation to match *T*_a_ and restricting regulation to the body rather than a large air space around the body. In all, the transition from an endogenous, metabolic existence to exploitation of exogenously powered climate control in minimalist or modern dwellings increases energy costs 9–28 fold.

Addressing Bateson’s [11] three categories, we thus find that the human transition from regulation (i.e., thermoregulating bodies with endogenous fuels) to extraregulation (i.e., thermoregulating dwellings with exogenous fuels) increases annual energy costs 9–28 fold, above and beyond the 15–30 fold increase that characterizes the adjuster-to-regulator transition [4]. These multiples are, in large part, measures of the contribution of thermoregulation to the carbon footprint of animal life. Far from emancipating humans from thermoregulation, occupancy of climate-controlled dwellings expands the cost of the thermoregulatory task, and humans have been paying this expanded cost for as long as they have used fire to heat occupied space. The costs of this extraregulation can be reduced through adaptive use of clothing insulation, restriction of per capita volume of thermoregulated air space, and climate-specific adjustment of dwelling insulation. Our analysis of city energetics provides little evidence of these energy conserving solutions in North America, and instead suggests a prioritization of power over efficiency [30], at vastly expanded scales [31], to meet the fundamental problem of temperature regulation in a variable environment.

## Materials and Methods

We approached the electric and natural-gas utilities of 39 mid-sized cities to obtain daily, city-wide usage data for an entire year, and here we report all six cities in which the primary energy sources are electricity and natural gas, and for which we were able to obtain data. Energy data at daily resolution were unavailable from the coldest-climate cities in North America because of widespread household reliance there on bulk deliveries of heating fuels. We calculated city heat production each calendar day as the sum of the heat equivalents of electric and natural-gas energy used (Dothan, Key West, and Kissimmee do not employ natural gas). For daily *T*_a_ we used the average of recorded maximum and minimum (Table S1).

For a comparison group of individual homeotherms, we chose eight species from a database of >200 published mammalian energy-*T*_a_ relationships identified via Web of Science keyword searches and literature cited in relevant publications. The eight species were selected from the whole database by the criteria of maximizing numbers of individual subjects studied while including ≤ 2 species from each mammalian order. Data plotted for each study (Fig. 1) were extracted from published graphs.

For both cities and individual mammal species, multi-phase regression (SAS 9.2, using an error structure accounting for both serial autocorrelation and heteroscedasticity) was used to identify *T*_LC_ and *T*_UC_ in the energy-*T*_a_ relationship. Candidate breakpoints were explored by initially fitting a 3-phase model. In regards the cities, for Ames, Dothan, and Kissimmee, the 3-phase model converged and had two significant breakpoints. For the other three cities, in which the 3-phase model did not converge or had one breakpoint where *P*>0.05, we subsequently fit a 2-phase model. In each case, the 2-phase model converged and had a significant breakpoint, which corresponded to *T*_LC_ (Flagstaff, Timmins) or *T*_UC_ (Key West). In regards the species of mammals, the 3-phase model was successful for the rabbit and shrew. In the six mammal species for which we needed to opt for a 2-phase model, we obtained a significant breakpoint corresponding to *T*_LC_ in five cases (cactus mouse, California mouse, Davy’s bat, glider, and vampire bat). In the sixth case, the fox, however, the 2-phase regression identified a V-shaped model with no thermoneutral zone (TNZ). Given that there was a 4°C data gap (24.5–28.5°C) in the vicinity of the identified breakpoint (29.6±1.7°C), we assumed the TNZ coincided approximately with this data gap. For all cities, RMR was estimated by averaging all measures of energy use within the TNZ identified by the multi-phase regression. For all mammal species, RMR was estimated in the same way, except that the fox RMR was estimated from the values on either side of the data gap. Equations for line segments below *T*_LC_ and above *T*_UC_ were calculated by linear least-squares regression. In all cases of both cities and mammal species, the r^2^ reported is the adjusted-r^2^ for the complete multi-phase regression.

For the meta-analysis of studies of naked humans, data were extracted from published graphs. In some cases, symbols in those graphs were plotted at *T*_a_s differing from those specified in the associated research-report text to avoid symbol overcrowding; in explicit cases of this type, *T*_a_s specified in the text were used for regression. Regression was forced through 37°C on the abscissa (see Fig. 1A).

For analysis of Jack London’s cabin, we determined dimensions and construction materials from historical photographs and descriptions [25]. We then summed estimated *U* (building conductance) values for all elements of the cabin envelope, plus an air infiltration factor, to obtain overall cabin *U* [26]–[28] (Table S3).

For decadal calculations of annual energy costs, we used energy-*T*_a_ relationships (Figs. 2–4) and *T*_a_ records (Table S1). We estimated the energy cost each day over a 10-year period (2000–2009) and calculated total annual cost from the average of all daily costs.

## Supporting Information

Table S1 **Sources of city** ***T*** **_a_ data.** Air temperature (*T*_a_) data were obtained from the records of these weather stations. For constructing Fig. 2, the *T*_a_ data used were for the same dates as the energy data. For decadal calculations of annual energy costs, the *T*_a_ data used were for all dates in the 10-year period 2000-2009.
(DOCX)

Table S2 **Derivation of energy-** ***T*** **_a_ plots for clothed people.** The insulation of clothing is typically expressed in clo units [23]. From the fundamental equations for clo calculations [20]–[21], the following equation can be derived to calculate the “comfort temperature,” *T*_comfort_ – the *T*_a_ at which a clothed, sitting, resting person will be comfortable (e.g., neither shivering nor sweating) – in an indoor environment where wind speed is 10 cm s^−1^ and relative humidity is <50%: *T*_comfort_  =  33 – 6.84 (*I*_Cl_ + 0.78), where *I*_Cl_ is the (unitless) clo value of the clothing. The equation yields a unitless value, which equals *T*_comfort_ in °C. To obtain the equations listed in the table, some of which (those for bolded clothing types) are plotted in Fig. 4B, we applied the first-order model in Fig. 1A, assuming *T*_LC_  =  *T*_comfort_, *T*_b_ = 37°C, and resting metabolic rate  =  104 W, the expected metabolic rate of a 178-cm-tall, 70-kg young adult male (body surface area  =  1.8 m^2^) [39] when area-specific metabolism is 1 met  =  50 kcal m^−2^ h^−1^ = 58 W m^−2^ [20].
(DOCX)

Table S3 **Estimation of conductance** ***C*** **of Jack London’s cabin.** Information on cabin construction and dimensions was obtained from published descriptions [25] and historical photographs. To minimize chances of error arising from unit conversions, calculations were carried out in the units customary in the United States building industry, and the final result was converted to W °C^−1^. Area-specific *U*, which is the inverse of the customary *R* value for building insulation, measures the thermal conductance of building materials. We used values of area-specific *U* and *R* from standard tabulations [26]–[28]. Air infiltration is inevitable, adding to the demand for heat to keep the inside at a fixed temperature. In the approach we took, following the protocol of physicist J. W. Shelton [28], the energy cost of counteracting infiltration was added to the energy cost of replacing heat lost by penetration through the building envelope to get an overall effective building conductance. Values for heat requirement calculated from this conductance and used in this paper (e.g., Fig. 4C) refer to heat that must be added to the interior of the cabin. If stove efficiency (percentage of heat released by burning transferred to the interior of the cabin as sensible heat) were about 40%, as seems likely [28], the heat equivalent of wood burned would have needed to be about 2.5 times greater than calculated from this conductance.
(DOCX)
