# Supplementary material for: City-Scale Expansion of Human Thermoregulatory Costs
Source: PLoS One. 2013 Oct 15;8(10):e76238. doi: 10.1371/journal.pone.0076238 (PMC3797062; doi:10.1371/journal.pone.0076238)
Supplement: Table S1 — Sources of city T a data. Air temperature (T a) data were obtained from the records of these weather stations. For constructing Fig. 2, the T a data used were for the same dates as the energy data. For decadal calculations of annual energy costs, the T a data used were for all dates in the 10-year period 2000-2009. (DOCX) [file pone.0076238.s001.docx]

**Table S1. Sources of city *T*_a_ data.**

| Property | City | | | | | |
| --- | --- | --- | --- | --- | --- | --- |
|  | Key West, Florida | Kissim-mee, Florida | Dothan, Alabama | Ames, Iowa | Flagstaff, Arizona | Timmins, Ontario |
| Recording agency | U.S. Natl Weather  Service | U.S. Natl Weather  Service | U.S. Natl Weather  Service | U.S. Natl Weather  Service | U.S. Natl Weather  Service | Environ- ment Canada |
| Station ID | GHCND: USW000 12836 | GHCND: USW000 12815 | GHCND: USW000 13839 | GHCND: USW000 94989 | GHCND: USW000 03103 | 6078285 |
| Station name | Key West Intl  Airport | Orlando Intl Airport | Dothan Regional Airport | Ames Munici- pal Airport | Flagstaff Pulliam Airport | Timmins Victor Power Airport |
| Station latitude (°N) | 24.6 | 28.4 | 31.3 | 42.0 | 35.1 | 48.6 |
| Station longitude  (°W) | 81.8 | 81.3 | 85.5 | 93.6 | 111.7 | 81.4 |

Air temperature (*T*_a_) data were obtained from the records of these weather stations. For constructing Fig. 2, the *T*_a_ data used were for the same dates as the energy data. For decadal calculations of annual energy costs, the *T*_a_ data used were for all dates in the 10-year period 2000-2009.
