# Supplementary material for: City-Scale Expansion of Human Thermoregulatory Costs
Source: PLoS One. 2013 Oct 15;8(10):e76238. doi: 10.1371/journal.pone.0076238 (PMC3797062; doi:10.1371/journal.pone.0076238)
Supplement: Table S2 — Derivation of energy- T a plots for clothed people. The insulation of clothing is typically expressed in clo units [23]. From the fundamental equations for clo calculations [20]–[21], the following equation can be derived to calculate the “comfort temperature,” T comfort – the T a at which a clothed, sitting, resting person will be comfortable (e.g., neither shivering nor sweating) – in an indoor environment where wind speed is 10 cm s−1 and relative humidity is <50%: T comfort = 33 – 6.84 (I Cl + 0.78), where I Cl is the (unitless) clo value of the clothing. The equation yields a unitless value, which equals T comfort in °C. To obtain the equations listed in the table, some of which (those for bolded clothing types) are plotted in Fig. 4B, we applied the first-order model in Fig. 1A, assuming T LC = T comfort, T b = 37°C, and resting metabolic rate = 104 W, the expected metabolic rate of a 178-cm-tall, 70-kg young adult male (body surface area = 1.8 m2) [39] when area-specific metabolism is 1 met = 50 kcal m−2 h−1 = 58 W m−2 [20]. (DOCX) [file pone.0076238.s002.docx]

**Table S2. Derivation of energy-*T*_a_ plots for clothed people.**

| Clothing | Clo | Source of clo value | *T*_comfort_  (°C) | Equation at *T*_a_ < *T*_comfort_  (*Y* = W person^-1^) |
| --- | --- | --- | --- | --- |
| **Full everyday clothing (long sleeves, long pants)** | 1 | Ref. 23 | 21 | *Y* = −6.5 (*T*_a_ − 37 ^o^C) |
| Ordinary full dress plus substantial overcoat and hat | 2.2 | Ref. 23 | 13 | *Y* = −4.3 (*T*_a_ − 37 ^o^C) |
| **All-wool winter sportswear** | 3.1 | Ref. 23 | 6 | *Y* = −3.4 (*T*_a_ − 37 ^o^C) |
| Polar clothing | 4.2 | Ref. 23 | −1 | *Y* = −2.7 (*T*_a_ − 37 ^o^C) |
| **Warmest Western clothing for active use –** calculated as midway between “polar clothing” and “maximal military operational clothing” | 5 |  | −7 | *Y* = −2.4 (*T*_a_ − 37 ^o^C) |
| Maximal military operational clothing | 6 | Ref. 22 | −13 | *Y* = −2.1 (*T*_a_ − 37 ^o^C) |
| **Maximal, traditional Inuit or Sami clothing: dual caribou pelts** | 12 | Ref. 22 | −54 | *Y* = −1.1 (*T*_a_ − 37 ^o^C) |

The insulation of clothing is typically expressed in clo units [23]. From the fundamental equations for clo calculations [20-21], the following equation can be derived to calculate the “comfort temperature,” *T*_comfort_ – the *T*_a_ at which a clothed, sitting, resting person will be comfortable (e.g., neither shivering nor sweating) – in an indoor environment where wind speed is 10 cm s^-1^ and relative humidity is < 50%: *T*_comfort_ = 33 – 6.84 (*I*_Cl_ + 0.78), where *I*_Cl_ is the (unitless) clo value of the clothing. The equation yields a unitless value, which equals *T*_comfort_ in °C. To obtain the equations listed in the table, some of which (those for bolded clothing types) are plotted in Fig. 4B, we applied the first-order model in Fig. 1A, assuming *T*_LC_ = *T*_comfort_, *T*_b_ = 37°C, and resting metabolic rate = 104 W, the expected metabolic rate of a 178-cm-tall, 70-kg young adult male (body surface area = 1.8 m^2^) [39] when area-specific metabolism is 1 met = 50 kcal m^-2^ h^-1^ = 58 W m^-2^ [20].
