# Supplementary material for: City-Scale Expansion of Human Thermoregulatory Costs
Source: PLoS One. 2013 Oct 15;8(10):e76238. doi: 10.1371/journal.pone.0076238 (PMC3797062; doi:10.1371/journal.pone.0076238)
Supplement: Table S3 — Estimation of conductance C of Jack London’s cabin. Information on cabin construction and dimensions was obtained from published descriptions [25] and historical photographs. To minimize chances of error arising from unit conversions, calculations were carried out in the units customary in the United States building industry, and the final result was converted to W °C−1. Area-specific U, which is the inverse of the customary R value for building insulation, measures the thermal conductance of building materials. We used values of area-specific U and R from standard tabulations [26]–[28]. Air infiltration is inevitable, adding to the demand for heat to keep the inside at a fixed temperature. In the approach we took, following the protocol of physicist J. W. Shelton [28], the energy cost of counteracting infiltration was added to the energy cost of replacing heat lost by penetration through the building envelope to get an overall effective building conductance. Values for heat requirement calculated from this conductance and used in this paper (e.g., Fig. 4C) refer to heat that must be added to the interior of the cabin. If stove efficiency (percentage of heat released by burning transferred to the interior of the cabin as sensible heat) were about 40%, as seems likely [28], the heat equivalent of wood burned would have needed to be about 2.5 times greater than calculated from this conductance. (DOCX) [file pone.0076238.s003.docx]

**Table S3. Estimation of conductance *C* of Jack London’s cabin.**

| Cabin part or process | Area  (ft^2^) | Area-specific *U*  (BTU ft^-2^ h^-1^ °F^-1^) | *U*  (BTU h^-1^ °F^-1^) |
| --- | --- | --- | --- |
| Walls  (logs, 9 inches thick) | 291 | 0.093 | 27.1 |
| Door  (wood planks, assumed 1.5 inches thick) | 11.3 | 0.56 | 6.3 |
| Windows  (glass) | 8.0 | 1.1 | 8.8 |
| Ceiling  (half logs, estimated 2.5 inches thick) | 150 | 0.33 | 49.5 |
| Floor  (soil) | 132 | 0.10 | 13.2 |
| Total *U* of cabin envelope  (This value measures the proclivity for heat penetration through the building envelope.) |  |  | 104.9 |
| Infiltration effect  (assumes 2 air changes per hour, thus 1984 ft^3^ h^-1^;  heat required: 0.018 BTU ft^-3^ °F^-1^) |  |  | 35.7 |
| Grand total: effective cabin conductance, *C* |  |  | 140.6 BTU h^-1^ °F^-1^  = 74.4 W °C^-1^ |

Information on cabin construction and dimensions was obtained from published descriptions [25] and historical photographs. To minimize chances of error arising from unit conversions, calculations were carried out in the units customary in the United States building industry, and the final result was converted to W °C^-1^. Area-specific *U*, which is the inverse of the customary *R* value for building insulation, measures the thermal conductance of building materials. We used values of area-specific *U* and *R* from standard tabulations [26-28]. Air infiltration is inevitable, adding to the demand for heat to keep the inside at a fixed temperature. In the approach we took, following the protocol of physicist J. W. Shelton [28], the energy cost of counteracting infiltration was added to the energy cost of replacing heat lost by penetration through the building envelope to get an overall effective building conductance. Values for heat requirement calculated from this conductance and used in this paper (e.g., Fig. 4C) refer to heat that must be added to the interior of the cabin. If stove efficiency (percentage of heat released by burning transferred to the interior of the cabin as sensible heat) were about 40%, as seems likely [28], the heat equivalent of wood burned would have needed to be about 2.5 times greater than calculated from this conductance.
